# JCBIE: a joint continual learning neural network for biomedical information extraction

**DOI:** 10.1186/s12859-022-05096-w

**Published:** 2022-12-19

**Authors:** Kai He, Rui Mao, Tieliang Gong, Erik Cambria, Chen Li

**Affiliations:** 1 School of Computer Science and Technology, Xi’an Jiaotong University, Xi’an, Shaanxi China; 2 Shaanxi Provincial Key Laboratory of Big Data Knowledge Engineering, Xi’an Jiaotong University, Xi’an, Shaanxi China; 3 National Engineering Lab for Big Data Analytics, Xi’an Jiaotong University, Xi’an, Shaanxi China; 4 School of Computer Science and Engineering, Nanyang Technological University, Singapore, Singapore

**Keywords:** Biomedical information extraction, Continual learning, Joint learning

## Abstract

Extracting knowledge from heterogeneous data sources is fundamental for the construction of structured biomedical knowledge graphs (BKGs), where entities and relations are represented as nodes and edges in the graphs, respectively. Previous biomedical knowledge extraction methods simply considered limited entity types and relations by using a task-specific training set, which is insufficient for large-scale BKGs development and downstream task applications in different scenarios. To alleviate this issue, we propose a joint continual learning biomedical information extraction (JCBIE) network to extract entities and relations from different biomedical information datasets. By empirically studying different joint learning and continual learning strategies, the proposed JCBIE can learn and expand different types of entities and relations from different datasets. JCBIE uses two separated encoders in joint-feature extraction, hence can effectively avoid the feature confusion problem comparing with using one hard-parameter sharing encoder. Specifically, it allows us to adopt entity augmented inputs to establish the interaction between named entity recognition and relation extraction. Finally, a novel evaluation mechanism is proposed for measuring cross-corpus generalization errors, which was ignored by traditional evaluation methods. Our empirical studies show that JCBIE achieves promising performance when continual learning strategy is adopted with multiple corpora.

## Introduction

The rapid increasing of biomedical knowledge from biomedical experiments and clinical practice provides considerable resources for biomedical information extraction [1–3]. Biomedical knowledge graphs (BKGs) organize biomedical entities and relations in the form of nodes and edges. Extracting entities, such as chemical/drug, protein/gene, and phenotype/disease, and their relations from unstructured text data is the foundation of developing large-scale biomedical BKGs [4–7]. In this work, we study Named Entity Recognition (NER) [8] and Relation Extraction (RE) [9] techniques to extract biomedical information. We further divide NER as entity span detection (SP) and entity type detection (ET) sub-tasks in our experiments for gaining better results in the RE task.

**Fig. 1:**
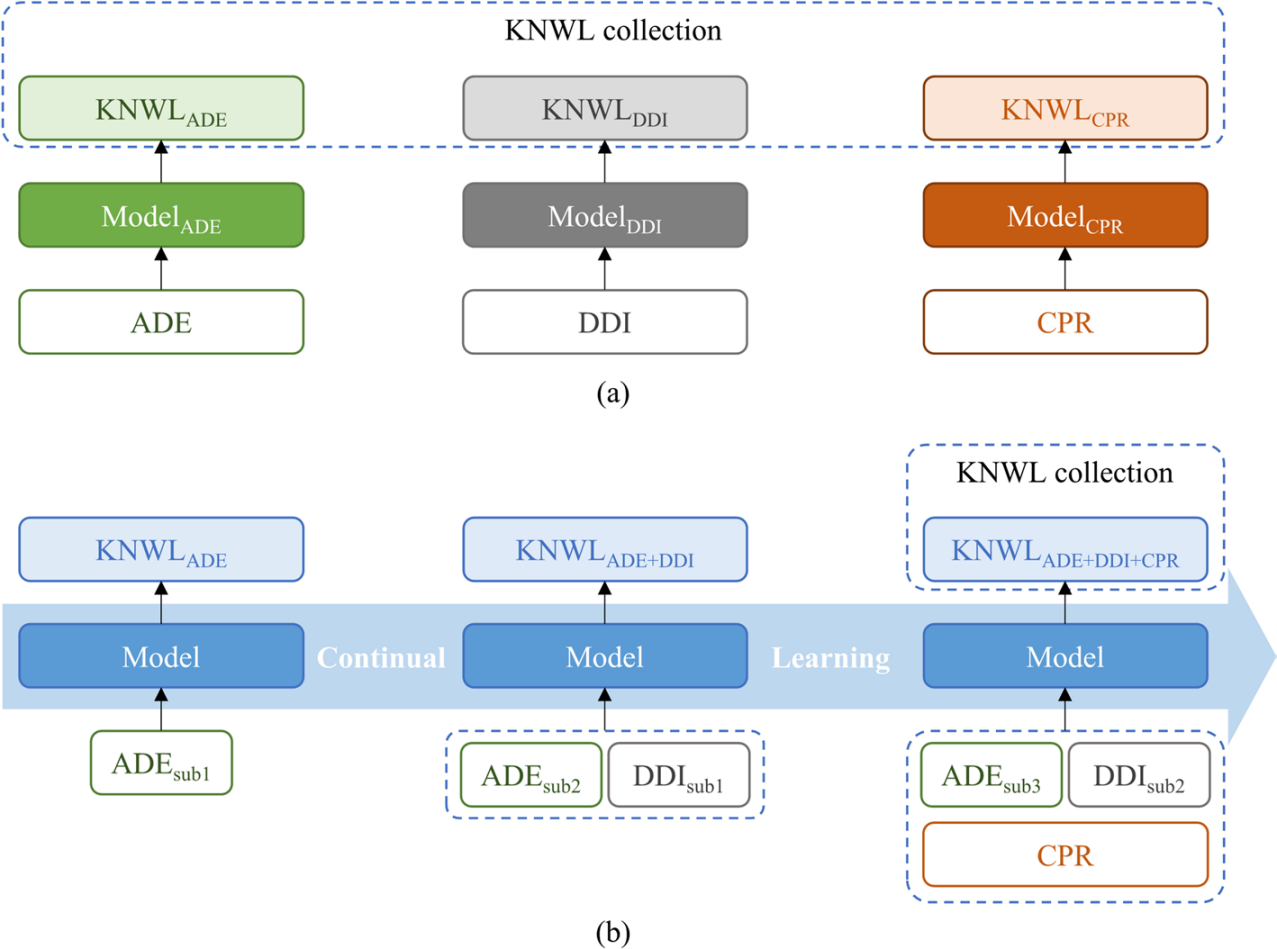
The difference between multiple-model learning and continual learning in biomedical information extraction. **a** Multi-models for extracting knowledge from multi-corpora. **b** A continual learning model. The input subscript with different numbers denotes different subsets in b. ADE, adverse drug events; DDI, drug–drug interaction; CPR, chemical protein reaction; KNWL, knowledge

Typical biomedical NER and RE tasks include the detecting of drug–drug interaction (DDI) [10, 11], adverse drug events (ADE) [12], chemical protein reaction (CPR) [13], protein–protein interaction (PPI) [14, 15], and mutation mining [16]. Each dataset only contains limited entity types and relation types, hence cannot support the understanding and inferring of entities and relations across tasks. For example, the ADE corpus only annotated drugs, diseases, and their interactions, and the CPR corpus only annotated the reaction relations and the entities of chemicals and proteins. However, sometimes we may require knowledge from both ADE and CPR to establish semantic interconnections between diseases of ADE and proteins of CPR by drug entities. Thus, traditional practices [17–19] developed multi-models to obtain knowledge from different datasets and learning tasks (see Fig. 1a). The limit of using multi-models is that the learning of common entity types (e.g., both ADE and CPR contain drug entities) cannot be shared across tasks and models. Besides, given a new corpus, extracting knowledge with multi-models is computationally expensive. In real-world practices, it is common to expand the size of an existing dataset, or learn new types of entities and relations from a new dataset over time. It is inconvenient to train a new model and maintain previous multiple trained models with every dataset update.

To solve the above problems, we propose a Joint Continual Learning Biomedical Information Extraction (JCBIE) network to jointly extract biomedical entities and relations based on a continual multi-corpora learning framework (see Fig. 1b). In order to learn new entity types and relation types over time by only one model, we use multi-head binary classifiers instead of a typical multi-class single-head classifier for ET and RE tasks. Thus, the size of pre-defined label set of entities and relations can be expanded by continually learning new datasets. Our method aims to support the constructions of extensible biomedical knowledge graphs with an extraction neural model.

We compare JCBIE (no-parameter sharing, multi-head classifier) with a traditional hard parameter-sharing and single-head classification method that was commonly used in current works [20–23], based on the same multi-corpora learning paradigm. JCBIE achieves an average gain of 2.77% micro-F1 scores over four different dataset fusion setups. We also examine the generalization abilities of traditional continual learning, multi-corpora learning, and our proposed continual multi-corpora learning approaches, based on different dataset feeding order setups and a different testing set. Our proposed learning paradigm yields average gains of 2.39% and 1.89% micro-F1 scores in a novel corpus-adaptation evaluation task over the two baseline learning paradigms, respectively.

We conduct systematic empirical studies for analyzing different variations in parameter-sharing mechanisms (Sect. 5.2), feature augmentation methods (Sect. 5.3), learning paradigms (Sect. 5.4), output-side classifier head types (Sect. 5.5), to answer the following questions: (1) What encoder parameter-sharing method is more suitable for learning SP, ET, and RE, simultaneously? (2) What feature augmentation method is more supportive for the RE task after identifying ET and SP of the NER task? (3) What is the difference between continual learning, multi-corpora learning, and continual multi-corpora learning? (4) Does a multi-class classifier (single-head) on the output side surpasses multiple binary classifiers (multi-head) in identifying multiple relation classes?

The contribution of this work can be summarized as twofold:

1. We propose a continual multi-corpora learning paradigm and an associated model with multi-head classifiers for ET and RE. The multi-head classifiers allow the model to expand the label vocabulary of entity types and relation types over time by feeding new datasets and introducing new label-oriented heads.
2. We conduct systematic empirical studies for analyzing different variations in model framework, feature augmentation methods, and learning paradigms. The results demonstrate the efficiency of the proposed method under different conditions.

**Table 1:** The example labels for exacting information from ADE, DDI and CPR

ADE	Input:	Two cases of mequitazine induced photosensitivity reactions							
	SP:	O	O	O	S	O	B	E	O
	ET:				Drug		Disease	Disease	
	RE:				ADE		ADE	ADE	
DDI	Input:	Thyroid may potentiate toxic effects of digitalis							
	SP:	S	O	O	O	O	O	S	O
	ET:	Drug						Drug	
	RE:	DDI						DDI	
CPR	Input:	… methyl rosmarinate activities against matrix metalloproteinase-1 …							
	SP:		B	E	O	O	O	S	
	ET:		Chemical	Chemical				Gene	
	RE:		CPR	CPR				CPR	

SP denotes entity spans, employing BIOES tagging scheme (Beginning-Inside-Outside-End-Single). ET, entity type; RE, relation extraction

## Related work

Joint extraction is a popular solution to biomedical datasets in DDI, ADE, CPR, and PPI [11–13, 15]. The basic assumption of joint extraction is that joint models can enhance the interactions between NER and RE [24], and alleviating the error propagation problem through sharing a common encoder [25–27]. Miwa and Bansal [20] firstly utilized a shared Bi-LSTM layer to encode input tokens, passing the word representations into NER and RE classifiers with dependency parsing features. Sun et al. [22] developed a joint extraction model based on a common graph convolutional network (GCN) encoder to perform a joint inference on entity types and relation types. These works are based on the assumption that the trained model is dataset-specific, which only needs to deal with the biomedical entity and relation types that have been defined in advance in a dataset. However, the data used to learn the same types of entities and relations are possibly supplemented over time in real-world practices. New entity types and relations are also gradually introduced in the biomedical research domain. Then, those dataset-specific models have to be retrained with new data and labels. Thus, a robust continual learning model is more fitting for the real-world applications.

The recent novel joint extraction research can be grouped into three sets. (1) The table filling strategy extracts information by labeling input tokens in a table. Miwa and Sasaki [28] utilized token lists of sentences to form rows and columns. Then, they extracted entities using the diagonal elements and classified relations with a lower triangular matrix of the table. Zhang et al. [29] integrated a global optimization technique and syntax information into the table-filling strategy to jointly train NER and RE. (2) Tagging scheme based methods jointly train NER and RE by designing customized tagging schemes. Zheng et al. [30] firstly proposed a novel tagging scheme that converts joint extraction to a tagging task. Yu et al. [31] decomposed the joint extraction into two sub-tasks. They first distinguished all head-entities, and then identifying tail-entities and relations jointly. (3) Seq2seq based methods regard NER and RE as a seq2seq generating task. Zeng et al. [32] proposed a CopyRE model, firstly introducing a Seq2Seq model for jointly extracting entities and relations to overcome the overlapped relation issue. Following, Zeng et al. [33] pointed out the CopyRE model could not distinguish head and tail entities. Then, they upgraded it to a CopyMTL model by adding a non-linear layer.

However, nearly all the above studies typically hypothesize that sharing parameters can provide better representations for joint NER and RE, failing to account for the differences between the two tasks. By utilizing different language models (LMs), model structures, and extraction strategies, these studies obtained state-of-the-art results. However, these methods did not properly control necessary variables for benchmarking. For example, a recent study [34] indicated that most joint extraction studies did not compare their joint methods with pipeline-based methods (e.g., comparing NER performance first, then RE) and compare different joint extraction methods with different pre-trained LMs. In such a condition, it is unsure whether empirical gains mainly come from joint model structures or different pre-trained LMs. Thus, we are motivated to conduct a systematic empirical study to demonstrate the utilities of different components of a typical NER and RE jointly learning model.

For continual learning, the main problem is catastrophic forgetting [35], which means a model forgets learnt knowledge after learning a new task. To alleviate this problem, ExtendNER [36] took the advantage of knowledge distillation to achieve continual NER tasks by transferring old knowledge in a teacher model to a new student model when new types occurred. Based on ExtendNER, L&R [37] supplemented synthetic samples which contained old type information to the knowledge distillation process, and found that such data replay process can boost performance for NER tasks. The research of [38] proposed a novel experimental framework that incorporated multiple tasks without explicit task identifiers. Also, this study proposed a benchmark and a new metric for continual learning, and concluded that replay models are better than memory-based solutions in a general continual learning setup. Different from the previous studies [36, 37], JCBIE only adopts a data replay method combined with multi-head classifiers to achieve continual learning and obtains satisfying results.

## Methodology

The learning target is formalized as joint NER and RE under continual learning setups. Unlike traditional approaches that consider NER as a single task in biomedical information extraction [23, 39], we divide the NER task as SP and ET tasks, respectively (seen Table 1). The SP task employs BIOES tagging scheme [30], where B, I, O, E, and S denote beginning, inside, outside, end, and single, respectively. Our JCBIE model continually learns SP, ET, and RE labels on token-level over different entity types and relation types from different datasets (ADE, DDI, and CPR).

We demonstrate the overall framework of JCBIE in Sect. 3.1. Our proposed method means to address the following challenges: Sect. 3.2. Efficient encoding for learning NER and RE tasks, simultaneously; Sect. 3.3. Efficient hidden state augmentation for learning RE; Sect. 3.4. A scalable classifier for continually learning new labels; Sect. 3.5. An efficient continual learning paradigm for learning dataset pipelines. To sum up, JCBIE employs non-parameter sharing encoders, entity marker augmented RE hidden state representations, multi-head classifiers, and a continual multi-corpora learning paradigm to fit the context of continual learning biomedical information extraction. The details of our proposed techniques (marked as $\bigstar$ in Sects. 3.2–3.5) and alternatives are shown in the following subsections.

### JCBIE

**Fig. 2:**
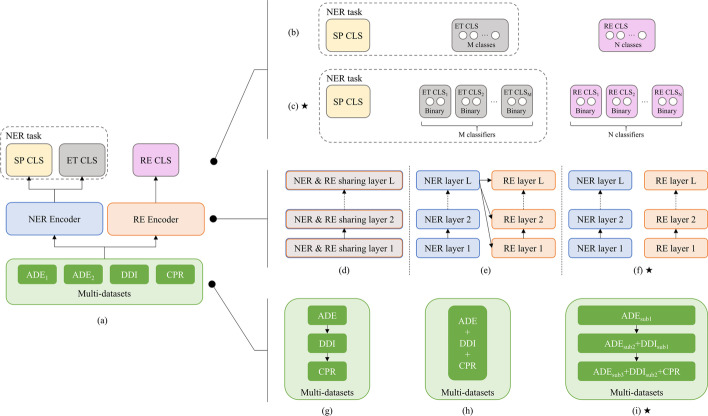
The framework and component variations for jointly learning NER and RE. **a** The overall framework. **b** Single-head classifier. **c** Multi-head classifier. **d** Hard-parameter sharing encoder. **e** Soft-parameter sharing encoder. **f** no-parameter sharing encoder. **g** Continual learning multi-datasets. **h** Multi-corpora learning. **i** Continual multi-corpora learning. Figure indices with $\bigstar$ (**c**, **f**, **i**) denote the proposed methods in JCBIE. The same components have the same color. SP denotes entity span; ET denotes entity type; CLS denotes classifier; ADE, DDI, and CPR denote different datasets, containing different entity types and relation types

As seen in Fig. 2a, JCBIE includes five technical components, namely Bio-BERT [40] based NER and RE encoders, SP, ET, and RE classifiers. In the training process, SP, ET, and RE are trained, simultaneously. In the inferring process, ET prediction is conditioned on SP results, and the relation prediction of two entities (RE) is conditioned on SP and ET results.

Given an input sentence $sent=\{x_1, x_2, ... x_i, ... x_n\}$, where *sent* is randomly sampled from a used dataset, $x_i$ ($1 \le i \le n$) is a natural language token, and *n* is the length of *sent*, JCBIE employs two Bio-BERT encoders for NER and RE, respectively. The output hidden states of each encoder are given by

1 $$\begin{aligned} h_{i}^{NER}=Encoder^{NER}\left( x_i\right) \end{aligned}$$

2 $$\begin{aligned} h_{i}^{RE}=Encoder^{RE}\left( x_i\right) . \end{aligned}$$

Noticeably, $h_{i}^{NER} \in {\mathbb {R}}^{1 \times d}$ is used for learning SP and ET labels for each token in a *sent*. $h_{i}^{RE}$ has the same shape with $h_{i}^{NER}$. *d* is the dimension of hidden states. Next, we employ three two-layer feed-forward networks as the classifiers ($T^{SP}(\cdot ),T^{ET}(\cdot ),T^{RE}(\cdot )$) upon the encoders, where a predicted SP label (${\hat{y}}_{i}^{SP}$) is given by Eq. (3). $Y^{SP}$ ($y_{i}^{SP} \in Y^{SP}$) denotes the ground-truth span of multiple entity mentions ($[e1,e2,...,e_{j},...,e_{k},...]$) in a sentence. We define the span of an entity mention $e_j$ covers the token indices from $\xi _{j}$ to $\epsilon _{j}$.

3 $$\begin{aligned} {\hat{y}}_{i}^{SP} = T^{SP} (h_{i}^{NER}). \end{aligned}$$

Then, the ET prediction of $e_{j}$ (${\hat{y}}^{ET}_{e_j}$, where ${\hat{y}}^{ET}_{e_j} \in {\hat{Y}}^{ET}$) is given by

4 $$\begin{aligned} {\hat{y}}^{ET}_{e_j}=T^{ET} \left( \sum _{i=\xi _j}^{\epsilon _j} h_{i}^{NER} \right) . \end{aligned}$$

The predicted RE label (${\hat{y}}^{RE}_{e_j,e_k}$, where ${\hat{y}}^{RE}_{e_j,e_k} \in {\hat{Y}}^{RE}$) of two random paired entities ($e_j$ and $e_k$) is given by

5 $$\begin{aligned} {\hat{y}}^{RE}_{e_j,e_k}=T^{RE} \left( v^{RE}_{e_j,e_k}\right) . \end{aligned}$$

$v^{RE}_{e_j,e_k}$ denotes the joint vector representation of RE hidden states, co-responding to $e_j$ and $e_k$. We will show the details of $v^{RE}_{e_j,e_k}$ later (the proposed $v^{RE}_{e_j,e_k}$ is given by Eq. (11) in Sect. 3.3).

### Parameter sharing in encoding

Previous studies claimed that information extraction models can benefit from a sharing encoder, because common parameters can enhance interactions between NER and RE [23, 30]. These methods can be categorized as hard parameter sharing and soft parameter sharing. Besides, we propose a no parameter sharing method.

*Hard parameter sharing* As shown in Fig. 2d, NER and RE use a sharing encoder. The encoder learns the hidden states (parameters) for both NER and RE tasks across layers.

*Soft parameter sharing* As shown in Fig. 2e, NER and RE have their private encoders, while the parameters of the last layer (*L*) of the NER encoder are shared for the learning of RE encoder layers. We employ a cross-attention mechanism [41] to constrain the parameter sharing. The post-fusion hidden states (${\mathcal {H}}$) of layer *l* in the RE encoder are given by

6 $$\begin{aligned} {\mathcal {H}}^{RE}_{l} = Softmax\left( \frac{H^{RE}_{l} \cdot {H^{NER}_{L}}^{\mathrm{T}} }{\sqrt{d}} \right) H^{NER}_{L} \cdot H^{RE}_{l}. \end{aligned}$$

where $H^{NER} \in {\mathbb {R}}^{n \times d}$ and $H^{RE} \in {\mathbb {R}}^{n \times d}$ are representations of a *sent*, which come from their private encoders.

$\varvec{\bigstar }$ *No parameter sharing* As shown in Fig. 2f, it employs two separated encoders for NER and RE. There is no interaction between NER and RE, which is the proposed encoding method in JCBIE.

### RE hidden state augmentation

We develop four augmentation methods, fusing the output hidden states ($v^{RE}_{e_j,e_k}$, mentioned in Eq. (5)) of RE encoders with NER features to enhance the learning of RE.

*Vanilla augmentation* A vanilla RE hidden state augmentation method is to concatenate ([; ]) the sum of NER and RE hidden states, corresponding to the same entity mentions, e.g., $e_j$ and $e_k$.

7 $$\begin{aligned} v^{RE}_{e_j,e_k} = \left[ \sum _{i=\xi _j}^{\epsilon _j}{h_{i}^{RE}};\sum _{i'=\xi _k}^{\epsilon _k}{h_{i'}^{RE}}\right] , \end{aligned}$$

where $v^{RE}_{e_j,e_k} \in {\mathbb {R}}^{1 \times 2d}$, $\xi _j$ and $\epsilon _j$ denote the start and the end indices of $e_j$, respectively; $\xi _k$ and $\epsilon _k$ denote the start and the end indices of $e_k$.

*Additional entity type embedding augmentation* We employ an linear embedding layer ($Linear(\cdot )$) to learn the embedding representations (*emb*) of entity types as the additional RE hidden state augmentation, where ${emb}_{e_j} =Linear(y^{ET}_{e_j})$; ${emb}_{e_k} =Linear(y^{ET}_{e_k})$. In the RE training process, we use the true label ($y^{ET}_{e_j}$) of an entity type that corresponds to the entity span $e_j$. In the RE inferring process, we use the predicted entity type label (${\hat{y}}^{ET}_{e_j}$). Then, the augmented RE hidden states are given by the concatenation of entity type embeddings and the vanilla hidden state augmentation

8 $$\begin{aligned} v^{RE}_{e_j,e_k} = \left[ \sum _{i=\xi _j}^{\epsilon _j}{h_{i}^{RE}};{emb}_{e_j};\sum _{i'=\xi _k}^{\epsilon _k}{h_{i'}^{RE}};{emb}_{e_k}\right] , \end{aligned}$$

where $${emb}_{e} \in {\mathbb {R}}^{1 \times 50}$$ and $$v^{RE}_{e_j,e_k} \in {\mathbb {R}}^{1 \times (2*d+2*50)}$$.

*Additional entity type prototype augmentation* An entity-type prototype representation is given by the original Bio-BERT encoder ($BioBERT(\cdot )$) output before training. We first collect all entity mentions from the training set, and categorize the entity mentions according to their entity types. The set of entity mentions (${\mathcal {S}}$) with a specific entity type ($y^{ET}$) is defined as ${\mathcal {S}}^{y^{ET}} = [X^{y^{ET}}_{1}, X^{y^{ET}}_{2},...,X^{y^{ET}}_{t}]$, where *X* is a token of the entity mentions. Totally, *t* tokens in ${\mathcal {S}}$. Then, the prototype representation (*proto*) of an entity type ($y^{ET}$) is given by

9 $$\begin{aligned} proto^{y^{ET}}=\frac{1}{t}\sum _{q=1}^{t} Maxpooling(BioBERT(X_{q})), \end{aligned}$$

where $proto^{y^{ET}} \in {\mathbb {R}}^{1 \times 50}$. In the RE training process, we look up to the prototype representations ($proto^{y^{ET}_{e_j}}$ and $proto^{y^{ET}_{e_k}}$) of a pair of entity mentions ($e_j$ and $e_k$), based on their true entity type labels ($y^{ET}_{e_j}$ and $y^{ET}_{e_k}$). The augmented RE hidden states are given by

10 $$\begin{aligned} v^{RE}_{e_j,e_k} = \left[ \sum _{i=\xi _j}^{\epsilon _j}{h_{i}^{RE}};proto^{y^{ET}_{e_j}};\sum _{i'=\xi _k}^{\epsilon _k}{h_{i'}^{RE}};proto^{y^{ET}_{e_k}}\right] . \end{aligned}$$

In the RE inferring process, we use the predicted entity type labels (${\hat{y}}^{ET}_{e_j}$ and ${\hat{y}}^{ET}_{e_k}$) to obtain prototype representations of $e_j$ and $e_k$, instead of gold labels ($y^{ET}_{e_j}$ and $y^{ET}_{e_k}$).

$\varvec{\bigstar }$ *Entity marker augmentation* Inspired by a recent mask language model [42] and the work of [43], we augment the raw input sentence with extra special tokens (entity markers) to highlight the positions of entities and the entity types. For each entity mention ($e_j$) in type $y^{ET}_{e_j}$, a start marker $[y^{ET}_{e_j\_start}]$ and an end marker $[y^{ET}_{e_j\_end}]$ are introduced into the raw sentence before and after the mention $e_j$. The example of an augmented sentence is “$[Drug_{\_start}]$ Pravastatin $[Drug_{\_end}]$ is associated with $[Disease_{\_start}]$ myotonia $[Disease_{\_end}]$ in animals”. We concatenate the RE encoder output hidden states of start markers of two entity mentions ($e_j$ and $e_k$) as the RE hidden state augmentation

11 $$\begin{aligned} v^{RE}_{e_j,e_k}= \left[ h_{e_j marker}^{RE};h_{e_k marker}^{RE}\right] , \end{aligned}$$

where $v^{RE}_{e_j,e_k} \in {\mathbb {R}}^{1 \times 2d}$. In the training process, NER encoder that is used for SP and ET learning takes an original raw sentence as input. The RE encoder takes the sequence with markers as input, where the entity spans and types are obtained, based on their true labels. In the inferring process, we predict entity spans (SP) and types (ET) with a raw sentence first, then insert the markers according to the SP and ET predictions for RE predictions.

### Single-head and multi-head classifiers

*Single-head classifier* As shown in Fig. 2b (the ET and RE classifiers are in grey and pink, respectively), single-head classifiers have two separated classifiers to predict multiple classes for ET and RE, respectively. In ET classification, e.g., a single-head classifier projects the prediction space into the vocabulary size (M classes) of all ET in a dataset. Then, the loss (${\mathcal {L}}^{total}_{s}$) of a sing-head (*s*) based model is the weighted sum of the cross-entropy losses of SP, ET, and RE

12 $$\begin{aligned} {\mathcal {L}}^{total}_{s} = \alpha ^{SP}{\mathcal {L}}^{SP}_{s} + \alpha ^{ET}{\mathcal {L}}^{ET}_{s} + \alpha ^{RE}{\mathcal {L}}^{RE}_{s}, \end{aligned}$$

where $\alpha ^{SP}$,$\alpha ^{ET}$, and $\alpha ^{RE}$ are hyperparameters. The limit of using single-head classifier is that the vocabulary of predicted labels cannot be expand after training.

$\varvec{\bigstar }$ *Multi-head classifier* Inspired by prompt learning that uses multiple prompts to infer labels for different tasks [44], JCBIE employs multi-head classifiers for ET and RE to fit the context of continual learning that entity types (ET) and relations (RE) can be expanded over time. The SP of JCBIE still uses a single-head classifier, because the vocabulary of SP labels is defined by the BIOES tagging scheme, regardless of dataset domains. As seen in Fig. 2c, ET and RE have *M* and *N* binary classifiers, learning *M* entity types and *N* relations, respectively. In ET classification, e.g., each binary classifier classifies whether an entity mention belongs to a specific type. Thus, JCBIE can expand the vocabulary of predicted labels over time by learning new datasets with new binary classifiers. The loss (${\mathcal {L}}^{total}_{m}$) of a multi-head classifier (*m*) based model is given by

13 $$\begin{aligned} {\mathcal {L}}^{total}_{m} = \alpha ^{SP}{\mathcal {L}}^{SP}_{s} + \alpha ^{ET}\sum _{\iota =1}^{M}{\mathcal {L}}^{ET}_{m,\iota } + \alpha ^{RE}\sum _{\kappa =1}^{N}{\mathcal {L}}^{RE}_{m,\kappa }, \end{aligned}$$

where the binary classifiers employ cross-entropy losses. If there are more than two binary classifiers that predict positive, JCBIE will take the result from the most confident classifier as the final prediction.

**Table 2:** Statistics of the employed datasets

Corpus	Sent. count	Entity mention counts			Relations counts		
		Ch./Dr.	Ph./Di.	Pr./Ge.	ADE	DDI	CPR
Training set							
${\text {ADE}}_{1}$	800	969	1144	–	1171	–	–
${\text {ADE}}_{2}$	3418	4063	4585	–	5422	–	–
DDI	5002	13,276	–	–	–	3607	–
CPR	8471	11,369	–	12,572	–	–	6044
Total	17,691	29,677	5729	12,572	6593	3607	6044
Validation set							
${\text {ADE}}_{1}$	100	124	129	–	140	–	–
${\text {ADE}}_{2}$	427	493	592	–	667	–	–
DDI	557	1487	–	–	–	413	–
CPR	1022	1490	–	1385	–	–	694
Total	2106	3594	721	1385	807	413	694
Testing set							
${\text {ADE}}_{1}$	100	115	144	–	142	–	–
${\text {ADE}}_{2}$	427	506	597	–	732	–	–
DDI	543	1480	–	–	–	475	–
CPR	1117	1715	–	1520	–	–	1016
Total	2187	3816	741	1520	874	475	1016
Corpus-adaption evaluation							
${\text {ADE}}_{3}$	4638	9517	2334	–	4767	–	–

Ch./Dr., chemicals or drug; Ph./Di., phenotype or disease; Pr./Ge., protein or gene

### Continual multi-corpora learning paradigm

*Continual learning* In order to extract different knowledge from different corpora to develop large-scale BKGs, continual learning was commonly used by recent works [45, 46]. The corpora are organized as a pipeline style for model learning sequentially (see Fig. 2g). When learning a new corpus, the parameters of a continual learning model are initialized as the parameters that were given by the learning of the last corpus. Thus, the initialized model is supposed to have remembered previous knowledge. However, [47] argued that such a continual learning method may result in the catastrophic forgetting of previously learnt knowledge. We will verify this in the later experiments.

*Multi-corpora learning* The ideal situation for training a model is to prepare an annotated corpus that contains all domain information. The model can learn the real world distribution of data from the omnipotent corpus. However, such a condition does not exist. We hypothesize that the collection of our prepared datasets is omnipotent in reflecting the real world data distribution; We do not need additional data to process ADE, DDI, and CPR datasets in the future (Hypothesis 1). A model trained with the combination of shuffled datasets (see Fig. 2h) shows the upper bond of learning performance, based on Hypothesis 1. We will demonstrate this later in empirical studies (Sect. 5.4). We will also show the result when Hypothesis 1 does not hold.

$\varvec{\bigstar }$ *Continual multi-corpora learning* Actually, datasets are continually expending in a research domain. For example, ADE$_1$ [48], DDI [49], ADE$_2$ [50], CPR [13], and ADE$_3$ [12] were developed in 2012, 2013, 2017, 2017, and 2019, respectively.

We mean to use a continual multi-corpora learning paradigm to mitigate the bias of a model continually learning data distribution, improving the corpus-adaption capacity of the model (see Sect. 5.4 later). As seen in Fig. 2i, we use the portion of an early dataset, e.g., ADE (here, ${\text {ADE}}_1$ and ${\text {ADE}}_2$ are combined, termed ADE) subset 1 (${\text {ADE}}_{\mathrm{sub1}}$) to train the model at Step 1. Then, the combination of ${\text {ADE}}_{\mathrm{sub2}}$ and ${\text {DDI}}_{\mathrm{sub1}}$ is used to continually train the model in Step 2. Finally, the rest of ADE and DDI data (${\text {ADE}}_{\mathrm{sub3}}$ and ${\text {DDI}}_{\mathrm{sub2}}$) combines CPR data to continually train the model at Step 3. In our experiments, ADE is divided into three equal parts (${\text {ADE}}_{\mathrm{sub1}}$, ${\text {ADE}}_{\mathrm{sub2}}$, and ${\text {ADE}}_{\mathrm{sub3}}$). DDI dataset is divided into two equal parts (${\text {DDI}}_{\mathrm{sub1}}$, and ${\text {DDI}}_{\mathrm{sub2}}$).

## Experiment

### Datasets

Chemical/drug, protein/gene, and phenotype/disease are three fundamental entity type classes to form complicated BKGs. We choose four biomedical corpus, including ${\text {ADE}}_1$ [48], ${\text {ADE}}_2$ [50], DDI [49], and CPR [13] for normal training and testing, and using ${\text {ADE}}_3$ [12] for corpus-adaptation evaluation. These corpora contain compatible definitions for the above entity types and relations. Table 2 shows the statistics of each dataset.

For a better compatibility, we normalize the entity types and relations in different corpora. E.g., entity type “Drug” in the DDI corpus is described as “any chemical agent used in the treatment, cure, prevention, or diagnosis of a disease that has been approved for human use”. Another type is “Drug_n” which is defined as “any chemical agent that affects living organisms”. However, these two entity types are not differentiated in the CPR corpus. Thus, we normalized “Drug_n” as “Drug”. For relation normalization, the original DDI corpus varies four fine-grained DDI relations. We normalize them as the same one. Finally, the employed entity type labels are chemical/drug, protein/gene, and phenotype/disease. Three relation labels are ADE, DDI, and CPR.

### Evaluation and measure

The reported testing results are given by the model and the training epoch, which yields the best performance on the associated validation sets. All results are reported by a five-time running averaged micro-F1 measure, where RE results are the main measure. SP is regarded as a sequence-labeling task, in which all tokens are labeled for calculating micro-F1 (see Table 1). The performance of ET depends on the predictions of SP. Recognized entities from ET are counted as true-positive (TP), if both its boundary (from SP) and type are correct. If a gold entity is missing, it will be counted as a false-negative (FN) instance. If an entity with wrong boundary or type, it is counted as one false-positive (FP) instance. RE task depends on the SP and ET results, because the errors of SP and ET are propagated to the RE model. Only if two entities and related relation types are the exact same as gold labels is counted as TP in RE. Missing triples are counted as FN instances. If RE predicts a relation label that is not the same as the gold label, it is FP. When it comes to multi-corpus learning, we regard all data as one corpus for the measure of micro F1.

Additionally, we introduce a corpus-adaptation evolution task, which evaluates the generalization of a model in the continual learning context. ${\text {ADE}}_3$ is used to evaluate JCBIE after training on ${\text {ADE}}_{1}$, ${\text {ADE}}_{2}$, DDI and CPR. Noticeably, There are deviations in the annotation guidelines of these corpora. Their data sources are also different. Although a model has been well-trained by the corpora ${\text {ADE}}_{1}$ and ${\text {ADE}}_{2}$, e.g., its performance may drop in ${\text {ADE}}_{3}$. This evaluation aims at simulating real application scenarios. When a neural network tries to learn similar concepts with no exact definition (the problem also may be introduced by the different understanding from different annotators), how does the model perform with such huge noised data. The following results demonstrate that JCBIE can effectively alleviate the problem.

### Baseline

(1) *ExtendNER* [36] is a knowledge distillation-based framework, which transfers old knowledge from a teacher encoder into a new student encoder with an extended linear classifier. When ExtendNER needs to recognize new entity types, the parameters of teacher encoder layers are copied to initialize the new student encoder, and the linear classifier built on the top of the student encoder is expanded with the additional dimensions for the new entity types.

(2) *L &R* [37] is a two-stage framework, which consists of a learning stage and a reviewing stage. At the learning stage, L&R follows ExtendNER to distill old knowledge from a teacher model into a student model. At the reviewing stage, L&R generates synthetic samples with old entity types for jointly training, aiming to alleviate the inter-type confusion [51].

The original ExtendNER and L&R were designed only for NER, and we re-implement the methods for joint SP, ET, and RE tasks. When only $ADE_1$ is employed, ExtendNER, L&R, and Typical Joint Extraction are equal, because they do not start to distill at the first step. For L&R, it should notice that we randomly sample 20 instances rather than generating synthesized data in the reviewing stage. The reason is the reviewing stage of L&R was designed for only one NER task, and it is hard to ensure generate appropriate instances for joint SP, ET, and RE tasks.

(3) *Typical joint extraction* The above two studies are knowledge distill-based methods. Considering JCBIE are data replay-based method, we design another replay-based method named Typical Joint Extraction for more comprehensive comparison. According to the most recent works [20–23], a common practice about jointly extracting entity spans, entity types and the relations between two entities is based on a hard-parameter sharing encoder (Fig. 2d) and a single-head classifier (Fig. 2b). We compare our proposed no-parameter sharing (Fig. 2f) and multi-head classifier (Fig. 2c) with this baseline method. For a fair comparison, other variables e.g., pre-trained language models (Bio-BERT), multi-corpora learning learning paradigms (Fig. 2h), and datasets are controlled.

### Hyper-parameter setups

For all experiments, batch size is 8. Learning rate is 5e−4 for AdamW optimizer [52]. $\alpha _{SP},\alpha _{ET},\alpha _{RE}$ in Eqs. (12) and (13) are 0.4, 0.25, and 0.35, respectively. The dimension of *emb* is 50 in Eq. (8). The max pooling size of *proto* is also set to 50 in Eq. (10). We employ Bio-BERT-base.

**Table 3:** Comparison between typical joint extraction and JCBIE, based on a multi-corpora learning paradigm

Method\Dataset		$\mathrm {ADE_1}$	ADE	DDI + ADE	ADE + DDI + CPR	Avg.
ExtendNER	SP	82.56	86.64	88.24	86.35	85.95
	ET	84.79	87.99	89.85	84.45	86.77
	RE	68.70	76.94	77.50	68.20	72.84
L &R	SP	82.56	89.07	90.04	90.58	88.06
	ET	84.79	90.02	93.25	89.64	89.43
	RE	68.70	81.12	79.12	70.01	74.74
Typical joint extraction	SP	82.56	88.77	92.41	89.35	88.27
	ET	84.79	89.13	92.09	88.05	88.52
	RE	68.70	79.53	78.32	70.16	74.18
JCBIE	SP	87.80	89.17	91.98	91.12	**90.02**
	ET	87.77	89.65	92.07	90.38	**89.97**
	RE	74.18	80.56	80.09	72.97	**76.95**

The bold means the best results

The results are measured by micro-F1. NB: Without a subscript specification, ADE is the combination of ADE${_1}$ and ADE${_2}$. When only $ADE_1$ is employed, ExtendNER, L&R, and Typical Joint Extraction are equal, because they do not start to distill at the first step

**Table 4:** Comparison between different RE hidden state augmentations

Corpus	Vanilla	Entity marker	Entity type embedding	Entity type prototype
${\text {ADE}}_{1}$	74.61	74.18	72.78	**74.69**
ADE	79.37	**80.56**	80.25	79.66
DDI + ADE	79.58	**80.09**	79.88	78.57
ADE + DDI + CPR	71.83	**72.97**	71.51	71.43
Avg.	76.35	**76.95**	76.10	76.09

The bold means the best results

The results are measured by RE micro-F1

**Table 5:** Within-corpora evaluation by different learning paradigms

Corpus	Continual	Multi-corpora	Conti. multi-corp.
ADE	79.91	**80.56**	80.40
ADE-DDI	**80.32**	80.09	80.01
DDI-CPR-ADE	72.18	**72.97**	72.58
ADE-DDI-CPR	70.78	**72.97**	71.93
Avg.	75.80	**76.65**	76.23

The bold means the best results

The results are measured by RE micro-F1

**Table 6:** Cross-corpora evaluation by continual learning (CL), multi-corpora learning (ML), and continual multi-corpora learning (CML), single-head classifier (S), and multi-head classifier (M)

Training methods	CLS	ADE	DDI- ADE	DDI- CPR- ADE	ADE- DDI	ADE- DDI- CPR	Avg.
CL	S	29.99	33.69	31.95	1.04	0.01	19.34
	M	30.06	34.13	32.04	26.73	23.21	29.23
ML	S	29.41	28.88	25.62	28.88	25.62	27.68
	M	**30.76**	32.29	26.66	**32.29**	26.66	29.73
CML	S	25.21	36.46	27.08	20.13	22.51	26.28
	M	28.16	**38.95**	**33.83**	28.01	**29.18**	**31.63**
Avg.	S	28.20	33.01	28.22	16.68	16.05	24.43
	M	29.66	35.12	30.84	29.01	26.35	30.20

The bold means the best results

The performance is measured by RE micro-F1 on ADE$_{3}$ corpus

**Fig. 3:**
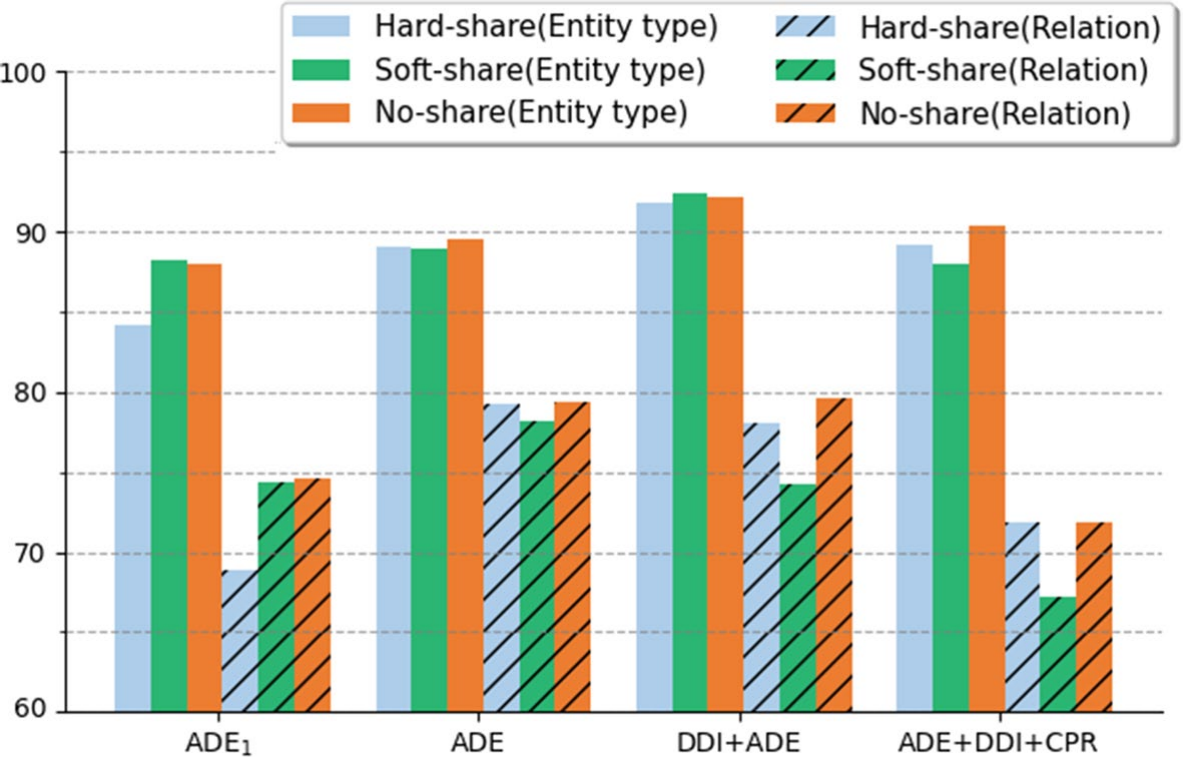
Comparison between different encoder parameter sharing methods. The performance is measured by micro-F1 on ET and RE tasks

## Results

In this section, we first demonstrate the improvements of JCBIE compared with a typical parameter sharing based joint extraction model and two other related baselines, then conducting empirical studies by comparing different encoding methods, RE hidden state augmentations, learning paradigms, classifiers, and finally discussing the NER tagging schemes and bottleneck factors in jointly learning NER and RE. 

### Proposed method versus baseline methods

In Table 3, all the compared models employ multi-corpora learning that shuffles data of all employed corpora as input. Compared with knowledge distillation-based ExtendNER and L&R, data replay-based methods (JCBIE and typical joint model) perform better. Besides, JCBIE performs better than the typical joint model in all four RE evaluations by different dataset combinations, yielding an average gain of 2.27%. JCBIE also achieves better performance on SP and ET tasks, yielding averaged gains of 1.75% and 1.45%, respectively. It shows the efficiency of JCBIE in a conventional multi-corpora learning paradigm overall.

### Different parameter sharing methods

Three types of encoding methods, including hard-parameter, soft-parameter, and no-parameter sharing are compared on ET and RE tasks in Fig. 3. For controlling variables, all compared models adopt multi-head classifiers (Fig. 2c) and multi-corpora learning (Fig. 2h). The results show that the soft-parameter sharing method is generally worse than the other two in RE task when more datasets, e.g., ADE, DDI + ADE, ADE + DDI + CPR are used for learning. Namely, the last hidden state of NER is not helpful for RE by cross-attention. This is probably because the ET information may mess up the RE learning when more labels are incorporated. By comparing hard-parameter sharing and no-parameter sharing setups, we find that no-parameter sharing outperforms hard-parameter sharing by 5.72% micro-F1 on ${\text {ADE}}_1$, while the performance of the two methods are close in the rest of dataset combinations. It shows that no-parameter sharing is particularly effective in single-corpus learning with the limited number of entities and relations. By comparing ET and RE, generally, RE task is more difficult because RE labels are more than that of ET.

### Different augmentation methods

Four different RE hidden state augmentation methods are compared in Table 4. All the results are given by a model armed with multi-head classifier (Fig. 2c), no-parameter sharing encoders (Fig. 2f), and multi-corpora learning (Fig. 2h). Apart from the ${\text {ADE}}_1$ evaluation task, adding entity markers is the optimal augmentation method for RE learning (76.95% micro-F1 on average). It helps a model to learn more diverse RE labels and tasks. For limited RE label learning in ${\text {ADE}}_1$, the vanilla augmentation method is slightly better. Establishing interactions between NER and RE from the input side (entity markers) is more useful than the fusing of hidden states on the encoder output side (other augmentation methods), because the Bio-BERT encoder delivers additional information fusion ability in modeling the interactions of two different tasks.

### Different training paradigms

We compare different learning paradigms, e.g., continual learning, multi-corpora learning and continual multi-corpora learning in two scenarios: (1) The testing and training sets are from the same corpora (within-corpora); (2) The testing and training sets are from different corpora (cross-corpora). The within-corpora evaluation analyses the ideal learning situation based on Hypothesis 1 (see Sect. 3.5). The cross-corpora evaluation is more close to the real-world situation, where Hypothesis 1 does not hold. The within-corpora evaluation is based on a model that has single-head classifiers (Fig. 2b), no-parameter sharing encoders (Fig. 2f) and entity marker augmentation methods. In the cross-domain evaluation task, we control encoder and augmentation methods, comparing classifier types (single-head and multi-head) and different learning paradigms. The dataset feeding pipeline in continual learning and continual multi-corpora learning is ordered. The datasets in multi-corpora learning is disordered, because all the datasets are combined as a whole dataset for training and testing.

As seen in Table 5, the three data learning paradigms yield similar performance, based on Hypothesis 1 and within-corpora evaluation. The multi-corpora learning achieves the highest micro-F1 across the four dataset setups, because it uses all datasets at once, learning the data distribution globally. The average gap between multi-corpora and continual multi-corpora learning paradigms is just 0.41%. It shows that continual multi-corpora learning also achieves comparable performance, based on Hypothesis 1.

In Table 6, we use an independent evaluation dataset (${\text {ADE}}_3$) for the cross-corpora evaluation to evaluate the corpus-adaptation ability of different learning paradigms and classifier types when Hypothesis 1 does not hold. By comparing different learning paradigms, multi-head classifier-based continual multi-corpora learning achieves the highest micro-F1 on average (31.63%), outperforming other learning paradigms by at least 1.9%. This shows that our proposed continual multi-corpora learning method tasks the complementary strength of continual learning and multi-corpora learning in cross-corpora evaluation. In contrast, continual learning models suffer catastrophic forgetting and tend to fit the last feeding corpus. E.g., when models are evaluated by ${\text {ADE}}_3$, they always perform better, if ADE is trained lastly (see the results in DDI-ADE vs. ADE-DDI; DDI-CPR-ADE vs. ADE-DDI-CPR). This observed phenomena is consistent with the study of [53].

Noticeably, micro-F1 values in cross-corpora evaluation in Table 6 are lower than within-corpora evaluation in Table 5. We list two major reasons here. Firstly, the boundaries between biomedical entities and other tokens are indistinguishable. E.g., “3-[(2-methyl-1, 3-thiazol-4-yl) ethynyl] pyridine” and “1-methyl-4-phenyl-1,2,3,6-tetrahydropyridine” are two drug entities in our data. Recognizing such entities without special training data is challenging. Secondly, certain annotation deviations exist in different corpora due to different annotation guidelines. For example, all kinds of inhibitors are regarded as Drug entity in ${\text {ADE}}_3$, but not in ${\text {ADE}}_1$ and ${\text {ADE}}_2$. Different genres can also lead to different performance for a supervised learning model [54].

### Single-head versus multi-head classifiers

We demonstrate the advantage of using multi-head classifiers based on different learning paradigms. As seen in Table 6, a multi-head classifier brings extra gains across all learning paradigms. This clearly demonstrates that a multi-head classifier surpasses a single-head classifier in cross-corpora evaluation. Multi-head classifiers also mitigate the impact of dataset-stream orders, reducing the gap between “DDI-CPR-ADE” (S: 28.22%, M: 30.84%) and “ADE-DDI-CPR” (S: 16.05%, M: 26.35%), e.g., from 12.17% to 4.49% on average. Thus, multi-head classifiers are more fitting for continual learning than single-head classifiers in robustness.

**Table 7:** Model performance on each corpus, measured by micro-F1

Corpus	NER	SP	ET	${\text {ET}}^+$	RE	${\text {RE}}^+$
${\text {ADE}}_1$	86.58	88.40	87.16	99.82	72.14	91.75
${\text {ADE}}_2$	90.66	92.18	90.72	99.81	83.37	98.64
DDI	95.48	96.74	96.42	100	80.00	84.26
CPR	88.58	90.59	89.19	97.97	65.36	74.27
Avg.	90.32	91.98	90.87	99.40	75.22	87.23

NB: NER means SP and ET labels are combined as a single label. ET and ET$^{+}$ denote the ET predictions depending on SP-predicted labels and gold labels, respectively. RE and RE$^{+}$ denote the RE predictions depending on SP and ET predicted labels and gold labels, respectively

## Discussion

In this section, we discuss (1) the impact of different NER annotation methods (united and separated tags), and (2) the impact of SP and ET errors on RE (bottleneck factors). We train JCBIE on ADE$_1$, ADE$_2$, DDI, and CPR datasets, individually. The JCBIE model is based on multi-head classifiers (Fig. 2c), no-parameter sharing encoders (Fig. 2f), and entity marker augmentation. The experiments do not involve continual learning and multi-corpora learning.

Traditional NER tagging scheme denotes both entity position and type information with a united label, such as “B_location, I_location, and E_location” [55, 56]. In contrast, we divide the NER label system as two separated SP and ET labels (see Table 1 for examples). In the inferring process, the ET prediction is conditioned on the SP results, which introduces an additional inference step. However, such a modification can reduce the label types in each task, improving model performance. Additionally, accurate predictions of SP and ET can improve the final predictions of RE, because the positions of entity markers are given by SP. The types of entity markers are given by ET. As seen in Table 7, by comparing ET and NER columns, JCBIE yields better performance in identifying entity types and positions based on SP-ET separated tagging scheme (90.87% micro-F1 on average) than the model trained with the traditional NER united tagging scheme (90.32%).

On the other hand, the errors introduced in SP and ET finally lower the RE performance. We first evaluate the error impacts of SP on ET. The ET$^{+}$ column in Table 7 shows the ET performance based on gold SP labels. By comparing ET and ET$^{+}$, we observe a drop of 8.53% in micro-F1 on average. ET$^{+}$ yielding 99.4% average micro-F1 highlights that the SP task performance is the bottleneck factor in NER task. We will explore a more accurate method for SP learning in the future. By using gold SP and gold ET labels, we observe RE$^{+}$ achieves 87.23%, exceeding RE by 12.01% on average. It shows that RE task is difficult. Although entity types and spans can be perfectly identified, there is still a huge space for improving RE performance. Thus, we will fuse additional knowledge for improving RE identification upon SP and ET in future work.

## Conclusion and future work

This paper explores JCBIE, jointly and continually learning biomedical information extraction from different corpora. We aim at establishing a more general biomedical information extraction neural network with continual learning ability. The ultimate goal is to get rid of limited entity types and relations to extract more knowledge, improving the generalization ability of a model. There are three summing-ups: Firstly, using two separated encoders without parameter sharing is better than using a hard-parameter sharing encoder or soft-parameter sharing encoders in learning NER and RE tasks; Secondly, apart from the ability of continually learning new entity types and relations, multi-head classifiers can also deliver better generalization on a new dataset; Finally, the dataset feeding orders have impacts on a cross-corpora inferring model. Using continual multi-corpora learning paradigm can somewhat mitigate the impacts, yielding robust performance.

In the future, we would further explore how to enhance the ability to continual learning. For example, utilizing a distillation-based method [36, 37] to transfer knowledge or using fuzzy clustering [57, 58] to filter features are both promising technologies to improve model performance. Besides, data replay-based continual learning is limited when previous data cannot access. We also try to explore methods that totally need no previous data while still can keep promising performance.

## Data Availability

The datasets and codes used during the current study are available from GitHub at https://github.com/KaiHe-better/JCBIE.git.

## Abbreviations

JCBIE: Joint continual learning biomedical information extraction
NER: Named entity recognition
RE: Relation extraction
SP: Entity span detection
ET: Entity type detection
DDI: Drug–drug interaction
ADE: Adverse drug events
CPR: Chemical protein reaction
PPI: Protein–protein interaction
LM: Language models
